# Technology Acceptance of a Machine Learning Algorithm Predicting Delirium in a Clinical Setting: a Mixed-Methods Study

**DOI:** 10.1007/s10916-021-01727-6

**Published:** 2021-03-01

**Authors:** Stefanie Jauk, Diether Kramer, Alexander Avian, Andrea Berghold, Werner Leodolter, Stefan Schulz

**Affiliations:** 1 Steiermärkische Krankenanstaltengesellschaft m.b.H. (KAGes), Information and Process Management, Graz, Austria; 2 Institute for Medical Informatics, Statistics and Documentation, Medical University of Graz, Auenbruggerplatz 2, 8036 Graz, Austria

**Keywords:** Clinical decision support, Delirium, Machine learning, Predictive modelling, Risk management, Technology acceptance model

## Abstract

Early identification of patients with life-threatening risks such as delirium is crucial in order to initiate preventive actions as quickly as possible. Despite intense research on machine learning for the prediction of clinical outcomes, the acceptance of the integration of such complex models in clinical routine remains unclear. The aim of this study was to evaluate user acceptance of an already implemented machine learning-based application predicting the risk of delirium for in-patients. We applied a mixed methods design to collect opinions and concerns from health care professionals including physicians and nurses who regularly used the application. The evaluation was framed by the Technology Acceptance Model assessing perceived ease of use, perceived usefulness, actual system use and output quality of the application. Questionnaire results from 47 nurses and physicians as well as qualitative results of four expert group meetings rated the overall usefulness of the delirium prediction positively. For healthcare professionals, the visualization and presented information was understandable, the application was easy to use and the additional information for delirium management was appreciated. The application did not increase their workload, but the actual system use was still low during the pilot study. Our study provides insights into the user acceptance of a machine learning-based application supporting delirium management in hospitals. In order to improve quality and safety in healthcare, computerized decision support should predict actionable events and be highly accepted by users.

**Supplementary Information:**

The online version contains supplementary material available at 10.1007/s10916-021-01727-6.

## Introduction

Artificial intelligence (AI) and particularly machine learning (ML) for supporting healthcare have been a constant in medical informatics research over decades [1, 2]. Health-related prediction modelling has gained much attention since well-known companies have been developing prediction models for different clinical outcomes [3]. This has given rise to various prediction models with high predictive performance in retrospective data sets. However, few of these models have ever been adopted to support healthcare professionals in clinical routine [4, 5].

Several barriers and concerns have been raised for the implementation of ML-based predictive models in clinical decision support systems [5–8]. As the final decision is always the responsibility of the user, it is crucial to open the often criticized black box of ML decisions so that healthcare professionals can detect bias or error [9].

While the simplicity of a system and education tailored to its use facilitate the uptake of a new technology, increasing workload and threats to the doctor/nurse-patient relationship might hinder it [10]. The fear of losing control over decision-making is a potential barrier [11], and alerts and recommendations might be ignored by clinicians if they are overwhelmed by them [12].

Two recent studies reported on the acceptance of ML-based applications by clinicians. Brennan et al. [13] evaluated the application MySurgeryRisk [14] in a clinical setting and compared the judgment of clinicians with the algorithm’s prediction of postoperative complications. Although physicians’ risk assessment significantly improved after interaction with the algorithm, only five out of ten physicians reported that the application helped them in decision-making. Five physicians reported that they would use the application for counselling patients preoperatively, and eight found it easy to use.

Ginestra et al. [15] assessed clinical perceptions of the Early Warning System 2.0 [16], a tool that predicts sepsis in non-ICU patients. Two hundred eighty-seven nurses and physicians completed a survey after an alert by the system. Overall, physicians criticised missing transparency of relevant predictors, too late alerts and that the system triggered mostly for already known abnormalities.

We recently implemented an ML-based application predicting the occurrence of delirium in an Austrian hospital, and prospectively evaluated its performance in a routine clinical setting [17]. Delirium is a syndrome of acute confusional state with an acute decline of cognitive functioning [18]. Delirium patients have an increased risk of morbidity and mortality. High occurrence rates of delirium do not only increase length of stays and financial costs [19], but present a high burden for nursing. Identifying patients with highest risk is especially beneficial for nursing, because delirium can be prevented by non-pharmacological interventions [20, 21]. During a pilot study of seven months, the performance of the algorithm had achieved a specificity of 82% and a sensitivity of 74% [17].

As much as an algorithm excels in prospective prediction, it is crucial to know how users and domain experts perceive it. A well-known model for evaluating new technologies is the Technology Acceptance Model (TAM) [22, 23], often referred to as a gold standard for explaining IT acceptance [24]. Based on the theory of reasoned action [25], TAM assumes that a behavioural intention acts as best determinant for the *actual use* of an innovation in technology, influenced by *perceived ease of use* and *perceived usefulness* of an innovation. In the extended model TAM2, perceived usefulness is further influenced by several more factors including the *output quality* of the system, i.e. how well the system performs [26]. Validity and robustness of TAM have been shown for the field of healthcare [27], but minor adaptions of the items are recommended when evaluating health IT applications [24].

The overall goal of our study was to gain knowledge of the uptake, user acceptance and concerns regarding a ML-based prediction application designed to improve patient safety in a clinical setting. The evaluation targeted perceptions by healthcare professionals on the use case delirium prediction and included domain experts and users who had been using the application regularly in their daily work.

## Material and methods

### The delirium prediction application

Starting in spring 2018, the delirium prediction application has been implemented in a hospital of Steiermärkische Krankenanstaltengesellschaft (KAGes), the regional public care provider in Styria, Austria. Prior to implementation in the hospital information system (HIS), we had performed various training sessions for healthcare professionals and had promoted the application throughout all participating departments.

For every patient admitted to one of the departments, a random forest-based algorithm automatically predicts the delirium risk based on existing EHR data [17]. The predicted outcome is an ICD-10-GM (International Classification of Diseases – Tenth Revision – German Modification) coded diagnosis F05 (Delirium due to known physiological condition) or mentions of delirium in the text of a patient’s discharge summaries. In addition, domain experts stated the need to include a second model that predicts the diagnosis F10.4 (alcohol withdrawal delirium). Although this type of delirium is quite distinct from the condition coded by F05 in terms of aetiology and pathophysiology, experts found it crucial to include both types because of their similarity in signs, symptoms and consequences.

**Fig. 1:**
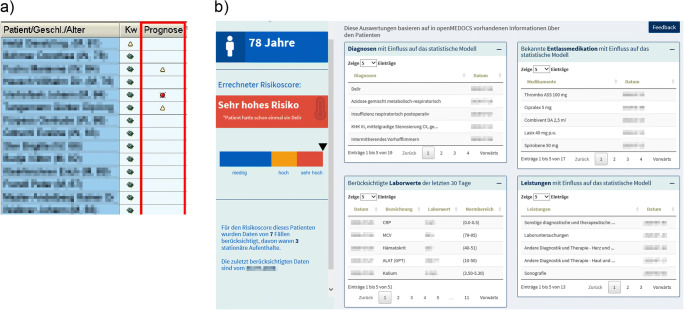
Visualization of the machine learning application in the hospital information system (**a**) and in a web application presenting patient specific features for prediction (**b**). For the sample patient, a very high risk of delirium is predicted (in red)

The algorithm predicts delirium risk with both models separately. Based on the higher risk score, every patient is stratified into a risk group: low risk, high risk or very high risk. An icon symbolizing the risk group is presented within the user interface of the HIS (Fig. 1a). With a click on the icon, a web application (Fig. 1b) opens up revealing details on the ML prediction supporting clinical reasoning [17, 28]: The application displays patient specific information used for modelling, e.g. ICD-10 codes, laboratory results or procedures. Predictors are ranked by (1) evidence-based risk factors of delirium known from literature and (2) the highest impact on the ML prediction using established feature importance functions.

### Study design

**Fig. 2:**
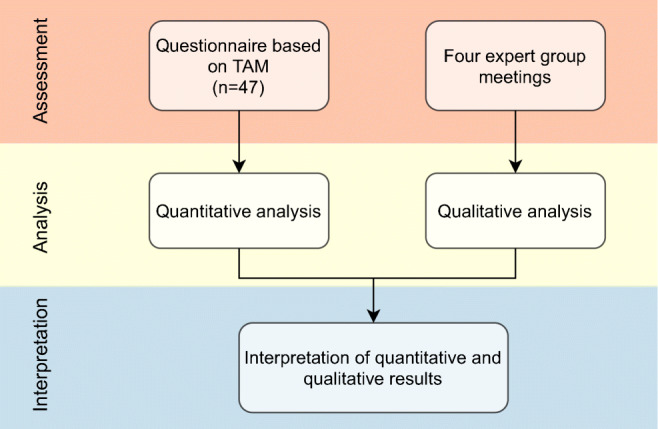
Convergent parallel study design using quantitative and qualitative methods including factors of the Technology Acceptance Model

In this study, we evaluated the delirium prediction application integrated in a HIS. This included the visualization in the user interface of the HIS (Fig. 1a) as well as a web application (Fig. 1b), which opens up from the HIS. We used a convergent parallel design for the mixed methods study (Fig. 2). For both quantitative and qualitative methods, TAM [22] constituted the evaluation framework. The factor output quality from TAM2 [26] was considered highly relevant for the application of complex machine learning models in healthcare and was thus added to the original TAM framework for evaluation.

In the qualitative assessment, two authors collected comments from healthcare professionals during four expert group meetings before and during the pilot phase. After the last meeting, one author assigned all comments to the factors of TAM – perceived ease of use, perceived usefulness, output quality and actual system use. Output quality was defined as the perceived correctness of delirium risk prediction. Besides sharing their experience with the application, the expert group suggested improvements for visualization in the HIS and new functionalities for the algorithm.

In the quantitative assessment, we evaluated the user acceptance of the application using questionnaires seven months after implementation. One author formulated items for the TAM factors based on original examples [26] and, as recommended in the literature [24], slightly adjusted to the context of healthcare and delirium prediction. After an expert discussion with two more authors, a total of 16 items were selected for the final questionnaire. A pilot test on understandability with two hospital staff members (not otherwise involved in the study) resulted in minor adoptions of item formulation. Responses for all items were measured using a five point Likert-type response scale (strongly disagree – strongly agree), apart from one item assessing the absolute frequency of use per month in numbers. The final questionnaire included 16 TAM items. User comments were assessed in a free text field at the end of the questionnaire (see Supplementary File Fig. S1).

Finally, quantitative and qualitative assessment results were interpreted in conjunction in order to obtain a detailed picture of the uptake of the application in the clinical setting.

### Participants

Printed questionnaires were distributed to five out of eight participating departments. Physicians and nurses from all levels of experience were encouraged to participate in the assessment, which was on a voluntary basis. We received completed questionnaires from ten out of 21 physicians (47.6%) and 37 out of 67 nurses (55.2%, see Table 1).

**Table 1:** Descriptive statistics for participants of the quantitative assessment (*n* = 47)

		**n**	**%**
Profession	Nurses	37	78.7
	Physicians	10	21.3
Sex	Male	14	29.8
	Female	33	70.2
		**Median**	**(Q1-Q3)**
Age, *years*		29	(26–42)

For the expert group meetings, the head of the department nominated experts from their field before the implementation. Depending on the clinical roster, up to five senior physicians and five ward nurses attended the meetings. Five ML engineers and IT professionals in charge of the HIS maintenance facilitated the usability engineering and algorithm improvement. Out of all 15 expert group members, eight members (53.3%) were male and seven (46.7%) female.

### Data analysis

**Fig. 3:**
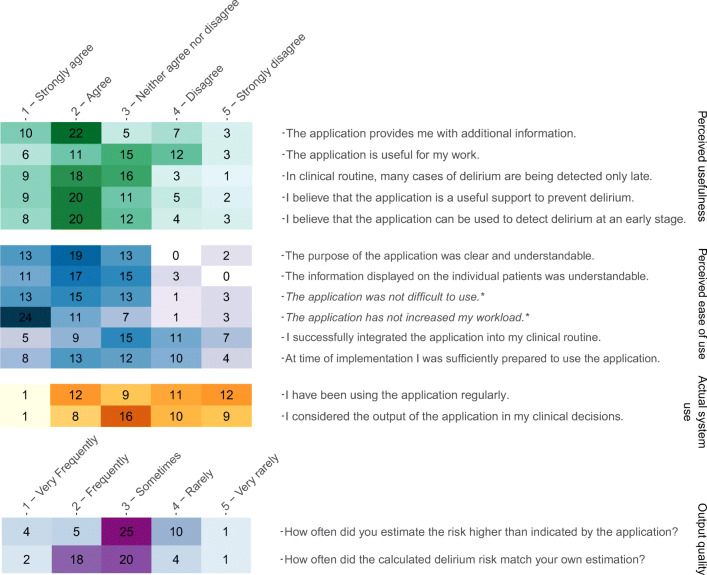
Heat map of 16 items assessing the technology acceptance of 47 users using four factors of the Technology Acceptance Model 2. Values are presented as absolute frequencies. The two items marked with asterisk were recoded for analysis: “*The application was difficult to use.”* (Original version) = “*The application was not difficult to use.”* (Recoded version); “*The application has increased my workload.”* (Original version) = “*The application has not increased my workload.”* (Recoded version)

All quantitative analyses were conducted in R Version 3.6. For all questionnaire items, heat maps facilitated the analysis of the results. For each participant the median was calculated for all item responses of each TAM factor, and then the mean of the medians of all participants was calculated for each factor. Two items measuring perceived ease of use had been formulated negatively and had to be recoded (see Fig. 3). In order to assess the internal consistency of the TAM factors in the questionnaire, we calculated the mean of the items for each factor and Cronbach’s alpha using the R package *ltm* [29] (see Supplementary File Table S2).

## Results

### Technology acceptance questionnaire

A heat map of the results from all 47 users on the questionnaire is shown in Fig. 3. Thirty-two users (68.1%) agreed or strongly agreed that the application provided them with additional information. Seven users (14.9%) did not believe that the application is a useful support for delirium prevention, and seven did not believe that the application can be used to detect delirium at an early stage. Opinions about the application’s usefulness for their own work were mixed: 17 users (36.2%) reported the application to be useful for their work, while 15 users (31.9%) did not find it useful.

Only two users did not find the purpose of the application understandable and three users reported that the presented information was not understandable. For 42 users (89.4%) the use of the application did not increase the workload. However, 18 users (38.3%) were not yet able to integrate the application successfully into their clinical routine, and 14 users (29.8%) reported that they were not sufficiently prepared to use the application at time of implementation.

Five users (10.6%) reported that the calculated delirium risk matched their own estimations only rarely or very rarely, and nine users (19.1%) reported that they frequently or very frequently estimated the risk higher than the application.

Considering actual system use, nine users (19.1%) strongly agreed or agreed that they considered the output of the application in their clinical decisions. Thirteen users (27.7%) reported that they had been using the application regularly, and the median for use per month was 3 times (min = 0, max = 20).

Overall, users rated the perceived ease of use and perceived usefulness rather positive, the output quality neutral, and the actual system use rather poor (see Table 2). Two users left a comment in the free text field. User A described the application as “an excellent instrument for delirium screening that allows managing prevention”. User B commented that “there was a more frequent use on the part of the physicians”.

**Table 2:** Mean responses to the factors of technology acceptance for the delirium prediction algorithm. Values were calculated for the median response of each participant on the factor. Questionnaire items were rated from 1 (positive/high) to 5 (negative/low)

	**Items (*n*)**	**Mean**	**SD**
Perceived usefulness	5	2.4	1.00
Perceived ease of use	6	2.3	0.79
Output quality	2	2.8	0.73
Actual system use	2	3.4	1.08

### Expert group meetings

#### Perceived usefulness

The consensus of the expert group on perceived usefulness was that the application offered a great support in early recognition of delirium risk patients and helped to reduce resources for screening.

*“The application gives good support – I am convinced of its usefulness.”*

*“Due to the delirium prediction application, we were already able to prevent the sliding into a strong delirium with simple interventions.”*

*“I see the application as a benefit, as we are able to reduce the time for delirium screening.”*

It provided support in the assessment of patients under sedation at admission, and it was used to confirm existing presumptions on delirium risk.

*“It is especially an added value if patients are not responsive during admission.”*

*“The prediction helps to corroborate my own estimation when seeing a patient.”*

*“Also, the prediction helps us when we are not quite sure about the delirium risk.”*

The application also supported the targeting of patients with a delirium diagnosis in a previous stay.

*“Especially patients with a diagnosis of delirium in the past are being targeted earlier now.”*

#### Perceived ease of use

The common impression for the perceived ease of use was highly positive. The expert group appreciated that there was no need of additional data entry and that the prediction was available within few seconds in the user interface of the HIS. As illustrated in Fig. 1a, high risk patients were presented with a yellow symbol and very high risk patients with a red symbol. The experts appreciated this.

*“I like the presentation with the traffic light symbol.”*

The visualization in the web application (Fig. 1b) sparked much enthusiasm, because it provided a comprehensive view of a patient supporting healthcare quality not connected to delirium prevention. However, during the first month the risk of delirium had been visualised using percentages. This was criticized by the experts, as their interpretation was not clear to them. As a solution, we replaced the percentages by a bar chart visualizing the three risk categories and an arrow indicating the location of a patient on the risk dimension.

*“The bar representing the range of delirium risk helps us to identify patients at the border to another risk group.”*

#### Output quality

Within the expert group, the predictive accuracy of the algorithm was perceived as very high.

*“The system has almost 100 % accuracy.”*

*“There are not too many patients in the very high risk group – it seems correct.”*

#### Actual system use

One senior physician raised concerns about the frequency of use among other physicians:

*“I absolutely want to continue with the application. Now the question is how to bring it closer to the users – many don’t know much about it yet.”*

Finally, there was a broad agreement of the expert group members to continue with the application in clinics, and to recommend the implementation of the algorithm to other hospital departments or hospital networks.

*“The application is successful. It should be continued in any case.”*

## Discussion

In this study, mixed methods with a convergent parallel study design were used to evaluate an ML-based application predicting delirium in in-patients from a user-centric perspective. The study provides significant insights to user acceptance with an ML application that uses EHR-based risk prediction to increase patient safety. A well-established theory, the Technology Acceptance Model [26], was used to frame the evaluation process and to guide the assessment of perceived usefulness, perceived ease of use, output quality and actual system use. A group of clinical experts provided regular feedback for qualitative analyses, and supported the improvement of visualization and algorithm functionality.

After seven months of implementation, the majority of users believed that the application was useful for the prevention of delirium or its early detection. They appreciated the visualization using yellow and red icons in the user interface of the HIS, and a detailed summary of the risk prediction in a web application. The automatic and fast prediction without the need of manual data entry presented a great value to them. However, not everyone was able to integrate the application into their clinical routine and the actual system use was low.

Studies of implemented ML applications are rare [4], and few studies have focused on the evaluation of user acceptance and technology uptake. A study of Brennan et al. [13] assessed user acceptance of an ML application as a secondary aim. However, their study sample was small and homogeneous including the feedback of ten physicians only. Ginestra et al. [15] included a bigger, heterogeneous sample, but the ML application was evaluated rather poorly due to missing transparency and late alerts.

In order to avoid a black-box scenario, we enhanced clinical reasoning using a web application presenting relevant features from ML-modelling (Fig. 1b). The presented information was understandable and the application provided users with additional information, e.g. highlighting previous diagnoses of delirium. Enabling interpretability and transparency of complex ML models facilitates clinical decision making and the appraisal of risk predictions, and thus remains an important task for ML developers [8].

Potential extra workload has been identified as a barrier of implementation [10], a result that might be essential for a successful uptake of ML-based applications in general. Users reported that the application did not increase their workload. However, further research is needed to determine whether a too high number of false positives might lead to additional preventive actions and increase the clinical workload unnecessarily.

Known barriers of hospital-based interventions such as staff workload and changes in roster [30] also limited our study. Questionnaires were kept as short as possible, and half of the staff members from five departments participated in the quantitative assessment. However, only 28% of the participants reported that they had regularly used the application, and the expert group concluded that more promotion and more training sessions were needed. Participation or non-response bias, e.g. people more positive towards an application are more likely to participate, might have affected the results [31].

A major limitation of our study is the questionnaire used for the quantitative assessment. Although TAM is extended by several factors in TAM2, we included only quality output. The need of a short and informative questionnaire limited number of items, and quality output seemed to be at highest importance to us. However, several factors are relevant for the usability of clinical decision support systems, which are not included in TAM nor TAM2 such as reaction speed or system errors [32]. Although the HIS of KAGes is known for its high stability, future studies should address the technical quality of the delirium prediction application integrated in the HIS.

Due to the limited sample size, psychometric analyses including factor analyses were not feasible and we analysed internal consistency for the TAM factors using Cronbach’s alpha only. The internal consistency was acceptable, but further analyses on the questionnaire are needed in future.

The aim of the expert group was to receive a broad feedback without restrictions to specific questions, and we did thus not conduct any structured interviews. Comments made by the expert group were documented and all of them could be assigned to the TAM factors chosen for evaluation. However, biases could occur for selecting questions and comments.

The last limitation to be mentioned is the rather short evaluation period restricted to one hospital only. Depending on clinical departments, staff members and predicted outcomes, feedback and evaluation results might vary, even with a stable performance of the underlying algorithm. Thus, ongoing monitoring and surveillance of the system as well as a continuous feedback loop with users is essential to determine the application’s usefulness and safety in the long term.

## Conclusion

The results of our study are unique, as we are among the first to implement a ML-based prediction application using electronic health records into clinical routine. The combination of quantitative and qualitative methods in the user-centric evaluation enriches our previously conducted evaluation of the performance of the algorithm during seven months of implementation. The high accuracy of the delirium prediction algorithm presented by us recently [17] is now supported by a positive technology acceptance by physicians and nurses. In future, similar applications providing reliable risk predictions and enhancing clinical reasoning will help targeting clinical resources for pharmacological and non-pharmacological preventive actions. We believe that the acceptance of a highly complex algorithm by healthcare professionals is an essential component for a successful implementation in a clinical setting. Without their belief in the usefulness of the application and their support during the whole implementation process, including the communication of existing opinions and concerns, an application is doomed to failure. Only ML algorithms that achieve high accuracy, predict actionable events and are highly accepted by healthcare professionals will be able to improve healthcare quality and hence patient safety.

## Supplementary Information

ESM 1 (PDF 445 kb)

## Data Availability

Data are available from the authors upon reasonable research proposals. In any case, permission of KAGes (Steiermärkische Krankenanstaltengesellschaft m.b.H., Stiftingtalstraße 4, 8010 Graz, Austria) is required.
