# Supplementary material for: Technology Acceptance of a Machine Learning Algorithm Predicting Delirium in a Clinical Setting: a Mixed-Methods Study
Source: J Med Syst. 2021 Mar 1;45(4):48. doi: 10.1007/s10916-021-01727-6 (PMC7921052; doi:10.1007/s10916-021-01727-6)
Supplement: Supplementary file 1 — (PDF 445 kb) [file 10916_2021_1727_MOESM1_ESM.pdf]

SUPPLEMENTARY FILE

Bitte markieren Sie für folgende Aussagen jene Kategorie, die am besten auf Sie zutrifft.

	Trifft nicht zu	Trifft wenig zu	Teils, teils	Trifft eher zu	Trifft sehr zu
Der Zweck des Tools war klar und verständlich.	☐	☐	☐	☐	☐
Das Tool ist nützlich für meine Arbeit.	☐	☐	☐	☐	☐
Ich war zu Beginn des Projekts ausreichend vorbereitet um das Tool verwenden zu können.	☐	☐	☐	☐	☐
Ich glaube, dass das Tool eine sinnvolle Unterstützung in der Delir-Prophylaxe ist.	☐	☐	☐	☐	☐
Durch das Tool stehen mir zusätzliche Informationen zur Verfügung.	☐	☐	☐	☐	☐
Das Tool war schwierig zu bedienen.	☐	☐	☐	☐	☐
Ich konnte die Verwendung des Tools gut in meinen klinischen Alltag integrieren.	☐	☐	☐	☐	☐
Durch das Tool ist meine Arbeitsbelastung gestiegen.	☐	☐	☐	☐	☐
Ich habe den Output des Tools in meine klinischen Entscheidungen miteinbezogen.	☐	☐	☐	☐	☐
Ich glaube, dass mithilfe des Tools ein Delir frühzeitig erkannt werden kann.	☐	☐	☐	☐	☐
Die angezeigten Informationen zu den einzelnen Patienten waren verständlich.	☐	☐	☐	☐	☐
In der täglichen Routine werden viele Delir-Fälle erst spät erkannt.	☐	☐	☐	☐	☐
Ich habe das Tool regelmäßig verwendet.	☐	☐	☐	☐	☐
Wie oft haben Sie das Delir-Prognose Tool verwendet (Ampel-Hinweis und/oder Detailanzeige)?	ca. _____ Mal pro Monat				
	**Sehr selten**	**Selten**	**Manch-mal**	**Häufig**	**Sehr häufig**
Wie häufig stimmte das berechnete Delir-Risiko mit Ihrer eigenen Einschätzung überein?	☐	☐	☐	☐	☐
Wie oft schätzten Sie selbst das Risiko höher als vom Tool angezeigt?	☐	☐	☐	☐	☐

Sonstige Kommentare/Anmerkungen:

Vielen Dank für Ihre Teilnahme!

Supplementary Fig. S1a. German version of the technology acceptance questionnaire used for quantitative assessment.

Please select for each statement the category which fits best for you.

	Strongly disagree	Disagree	Neither agree, nor disagree	Agree	Strongly agree
The purpose of the application was clear and understandable.	☐	☐	☐	☐	☐
The application is useful for my work.	☐	☐	☐	☐	☐
At time of implementation I was sufficiently prepared to use the application.	☐	☐	☐	☐	☐
I believe that the application is a useful support to prevent delirium.	☐	☐	☐	☐	☐
The application provides me with additional information.	☐	☐	☐	☐	☐
The application was difficult to use.*	☐	☐	☐	☐	☐
I successfully integrated the application into my clinical routine.	☐	☐	☐	☐	☐
The application has increased my workload.*	☐	☐	☐	☐	☐
I considered the output of the application in my clinical decisions.	☐	☐	☐	☐	☐
I believe that the application can be used to detect delirium at an early stage.	☐	☐	☐	☐	☐
The information displayed on the individual patients was understandable.	☐	☐	☐	☐	☐
In clinical routine, many cases of delirium are being detected only late.	☐	☐	☐	☐	☐
I have been using the application regularly.	☐	☐	☐	☐	☐
How often did you use the delirium prediction application? (Symbol in HIS and/or Web App?)	around __ times per month				
	**Very Rarely**	**Rarely**	**Some-times**	**Frequently**	**Very frequently**
How often did the presented delirium risk match your own risk estimation?	☐	☐	☐	☐	☐
How often did you estimate the risk to be higher than predicted by the application?	☐	☐	☐	☐	☐

Comments:

Thank you for your participation!

Supplementary Fig. S1b. Forward translated version of the technology acceptance questionnaire in English. The two items marked with asterisk were recoded for analysis later.

Supplementary Table S2. Internal concistency for the TAM factors used in the questionnaire. 95 % confidence intervals (CI) of Cronbach's alpha were calculated with the *ltm* package in R using boosting methods.

		Cronbach's alpha		
TAM factor	Items (n)	alpha	Lower CI	Upper CI
Perceived usefulness	5	0.76	0.650	0.837
Perceived ease of use	6	0.75	0.604	0.842
Output quality	2	0.66	0.070	0.876
Actual system use	2	0.85	0.743	0.918
